# Visual light flicker stimulation: enhancing alertness in sleep-deprived rats

**DOI:** 10.3389/fnins.2024.1415614

**Published:** 2024-06-05

**Authors:** Kun Wang, Kang Chen, Zilin Wei, Tianhui Wang, Aili Wei, Xiujie Gao, Yingkai Qin, Yingwen Zhu, Yi Ge, Bo Cui, Mengfu Zhu

**Affiliations:** ^1^Military Medical Sciences Academy, Tianjin, China; ^2^Medical Support Technology Research Department, Systems Engineering Institute, Tianjin, China; ^3^Tianjin Key Lab of Exercise Physiology and Sports Medicine, Tianjin University of Sport, Tianjin, China; ^4^Logistic Support Department of Central Military Commission, Beijing, China

**Keywords:** visual flicker, central fatigue, sleep deprivation, cortisol, serotonin

## Abstract

**Introduction:**

In the evolving field of neurophysiological research, visual light flicker stimulation is recognized as a promising non-invasive intervention for cognitive enhancement, particularly in sleep-deprived conditions.

**Methods:**

This study explored the effects of specific flicker frequencies (40 Hz and 20–30 Hz random flicker) on alertness recovery in sleep-deprived rats. We employed a multidisciplinary approach that included behavioral assessments with the Y-maze, in vivo electrophysiological recordings, and molecular analyses such as c-FOS immunohistochemistry and hormone level measurements.

**Results:**

Both 40 Hz and 20–30 Hz flicker significantly enhanced behavioral performance in the Y-maze test, suggesting an improvement in alertness. Neurophysiological data indicated activation of neural circuits in key brain areas like the thalamus and hippocampus. Additionally, flicker exposure normalized cortisol and serotonin levels, essential for stress response and mood regulation. Notably, increased c-FOS expression in brain regions related to alertness and cognitive functions suggested heightened neural activity.

**Discussion:**

These findings underscore the potential of light flicker stimulation not only to mitigate the effects of sleep deprivation but also to enhance cognitive functions. The results pave the way for future translational research into light-based therapies in human subjects, with possible implications for occupational health and cognitive ergonomics.

## 1 Introduction

Central fatigue, often associated with persistent sleep disturbances, manifests as a neuromuscular dysfunction with identifiable biochemical changes in the brain (Meeusen et al., 2006). In this context, light serves as a crucial modulator of non-visual functions, significantly enhancing alertness and cognitive performance. Studies have shown that specific light treatments, particularly involving flicker, are effective in increasing vigilance and improving overall cognitive task performance by influencing circadian rhythms and neural activity (Yuda et al., 2017; Yan et al., 2018; Hayano et al., 2021; Nie et al., 2021). Despite promising results, comprehensive understanding of the mechanisms through which flicker-induced light influences alertness and interacts with central fatigue remains sparse and warrants further investigation.

Light flicker is recognized for its ability to entrain cortical neural oscillations—the rhythmic fluctuations of electrical activity within the brain (Adaikkan et al., 2019; Adaikkan and Tsai, 2019; Tian et al., 2021). This process, facilitated by the transmission of flicker frequency data through the thalamocortical pathway, is essential for the generation of cerebral rhythms (Mathalon and Sohal, 2015; O’Reilly et al., 2021). Different stimulation frequencies are known to elicit varied cognitive responses, demonstrating the specificity of flicker effects (Vialatte et al., 2009; Notbohm et al., 2016). Notably, gamma rhythmic light flicker (either 40 Hz or 60 Hz) has been shown to non-invasively synchronize cortical gamma neural oscillations, potentially enhancing cognitive functions, such as learning and memory, thereby revealing the therapeutic promise of flicker stimuli (Singer et al., 2018; Yao et al., 2020; Lin et al., 2021; Venturino et al., 2021). Although exposure to light generally suppresses alpha, theta, and other lower-frequency neural activities—markers of drowsiness (Yokoi et al., 2003; Vandewalle et al., 2009; Wang et al., 2022), the specific impact of visual flicker stimuli on central fatigue and entrainment effects is less understood. Moreover, light influences fatigue and drowsiness via circadian rhythm mechanisms integral to our biological clock (Wong and Fernandez, 2021). For instance, exposure to morning light is known to boost alertness by stimulating the brain’s production of serotonin (5-hydroxytryptamine, 5-HT), which supports emotional well-being and physical activity. In contrast, the absence of light at night reduces cortisol production and promotes melatonin release from the pineal gland, thus decreasing alertness (Fisk et al., 2018). Chronic sleep deprivation disrupts these neuronal and hormonal processes, altering cortisol and 5-HT levels critical to the sleep/wake cycle (Saper et al., 2005; Kang et al., 2021). The potential of light flicker to recalibrate these hormonal levels and modulate central fatigue is an ongoing area of research.

Light flicker may modulate brain functions through two primary pathways: the induction of neural oscillations and the regulation of the circadian rhythm system. This study aims to explore the effects of random light flicker stimulation on the thalamus, a critical node in the neural circuitry involved in oscillatory brain dynamics. Additionally, this research compares the efficacy of 40 Hz flicker and 20–30 Hz random flicker in enhancing alertness and facilitating central fatigue recovery in sleep-deprived rats. Through this comparison, we hope to reveal how specific frequencies of light flicker can optimize cognitive functions and neural health, thereby providing a theoretical basis and practical guidelines for clinical applications.

## 2 Materials and methods

### 2.1 Animals and housing

The animal study was reviewed and approved by the Institutional Animal Care and Use Committee (IACUC) of the Chinese Academy of Military Medical Science. We used 32 male Sprague–Dawley rats, 8 weeks old, weighing 220–280 g each, obtained from Beijing HFK Bioscience Co., Ltd. These animals were accommodated in 15 cages (4–5 rats per cage) under standard conditions with a temperature of 20–26°C and relative humidity of 40–70%. The light/dark cycle was maintained at 12 h each, with lights on at 8:00 am. All rats had free access to food and water. After an acclimatization period of 7 days, 12 rats were selected for electrophysiological experiments. The remaining rats were randomly divided into four groups: a blank control group (Ctr, *n* = 5), a sleep deprivation group (Sleep deprivation, *n* = 5), a sleep deprivation +40 Hz visual flicker group (40 Hz, *n* = 5), and a sleep deprivation + random visual flicker group (Random, *n* = 5). Additionally, our adherence to the ARRIVE (Animal Research: Reporting of *In Vivo* Experiments) guidelines was meticulous, covering all aspects of animal handling, execution of experimental protocols, and documentation of findings.

### 2.2 Electrophysiological recording

The brain electrophysiology of rats was recorded, *n* = 12. The rats were anesthetized with a 2% sodium pentobarbital injection (40 mg/kg) administered intraabdominally. Their pain sensitivity was assessed through paw pinches. Following anesthesia, the rat’s head was shaved and securely fixed in a standard stereotaxic frame. A midsagittal incision was made in the scalp, and then a 3-mm square hole in the craniotomy window was carefully created. This hole was positioned 4 mm anterior to the Bergmann’s area and 4 mm to the right of the midline. Four skull screws were inserted into burr holes that had been drilled using a microdrill. Bone debris was then washed away using phosphate-buffered saline. After exposing the endocranium, a microwire array (Plexon, 2 × 2, with column and row spacing of 250 μm) was implanted into the thalamus. The reference and counter electrodes were subsequently connected to the ground bone screws on the skull using silver-coated copper wires. To insert the electrode array, a micro positioner was used, gradually advancing approximately 100 μm every 40 s until reaching a depth of 4,000 μm. Following a week of recovery, the neural acquisition processor (Plexon, Dallas, TX, United States) and its preamplifier were utilized to collect neural electrophysiological data (Jeczmien-Lazur et al., 2019). Light flicker was initiated at the 30th second of data recording, lasting for 2 min. Upon completion of the stimulation, data recording continued for 30 s.

### 2.3 Local field potential analysis

To analyze LFP data, we employed a multi-level fast Fourier transform technique via Neuroexplorer (Nex Technologies, United States). The data underwent filtering between 0 and 95 Hz using the Continuous Variables Digital Filter in the NeuroExplorer software. We produced a frequency-power curve using the Continuous Power Spectra function of the same software. Furthermore, the LFP frequency spectrum heatmap was crafted utilizing the Spectrogram function within NeuroExplorer.

### 2.4 Spike analysis

During electrophysiological studies, spikes that were twice as large as the standard deviation of the ambient noise were identified. We validated the accurate isolation of single units by observing interspike interval histograms. Using the Offline Sorter software (Nex Technologies, United States), waveforms extracted from the ongoing signal were categorized through both the Template Matching Method and Principal Component Analysis (PCA). Waveforms that displayed brief interspike intervals (less than 2 ms) were excluded. With the help of Neuroexplorer, we computed Peristimulus time histograms (PSTHs) with a bin size of 0.1 s to pinpoint light-responsive neuron units within the thalamus. Neurons were deemed light-responsive if their firing rate during light exposure surpassed the confidence limits of 95%.

### 2.5 Sleep deprivation model

**Figure 1:**
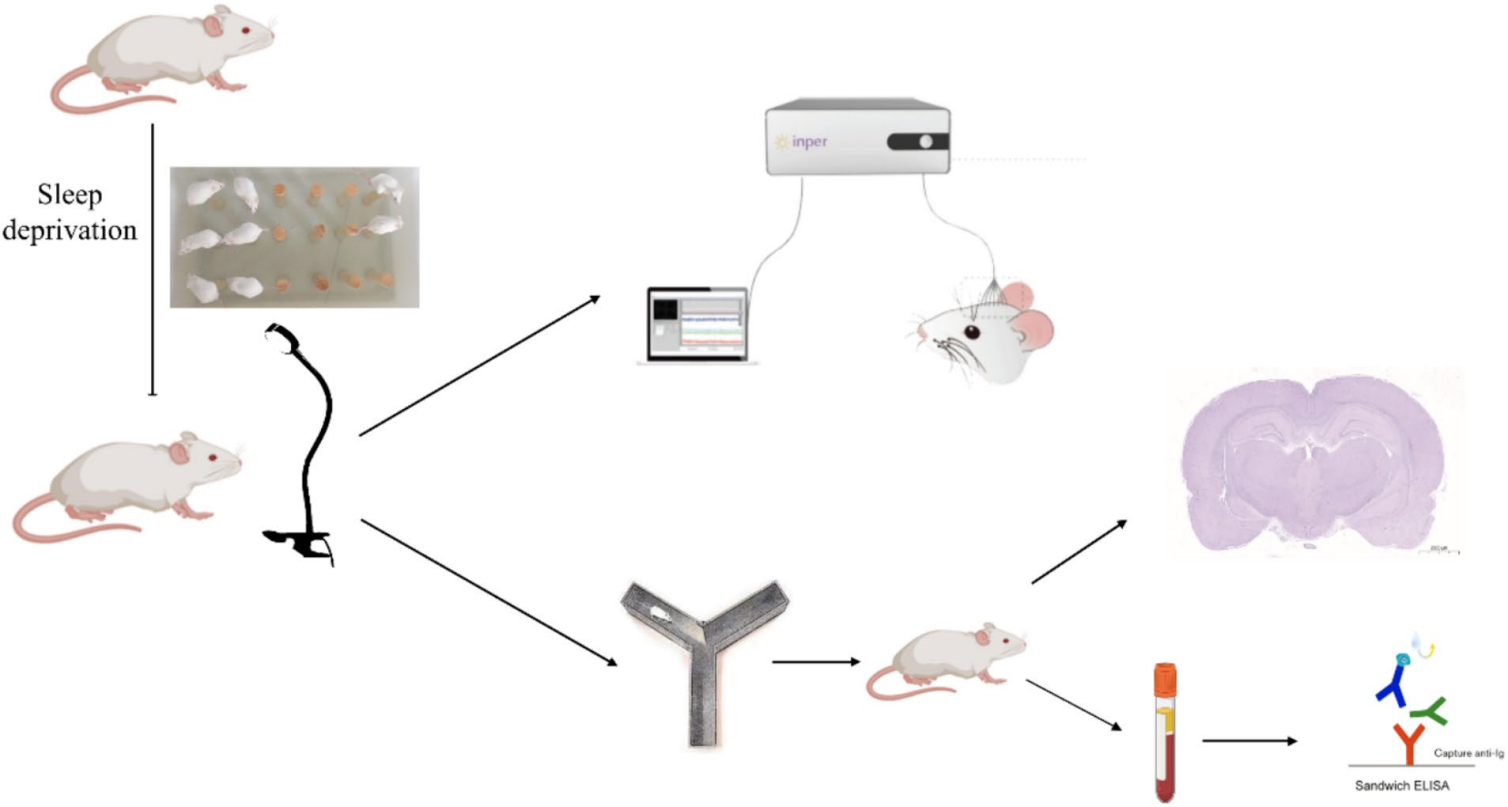
An experimental flowchart.

Rats underwent continuous sleep deprivation starting at 8 am for a duration of 72 h using a modified multiple-platform water bath method. Two water tanks were utilized in total, with 30 platforms positioned inside one of the tanks. Twenty rats were introduced into this water bath. Each rat had the freedom to jump from one platform to another. The water level in the bath was maintained at a height of 5 cm from the bottom. During the rapid eye movement (REM) sleep phase, a rat’s muscle tone changes, causing an imbalance. This would make the rat fall into the water, subsequently waking it up, and it would then climb back onto a platform to prevent drowning. To maintain hygiene, the water was replaced every 6 h throughout the experiment (Figure 1).

### 2.6 Visual light flicker exposure

Following sleep deprivation, the rats underwent light stimulation. We employed a sophisticated flickering light apparatus engineered for precise manipulation of light exposure parameters. This system, featuring a 30-W LED source (SEN-NO-HSL-39536, Shen Zhen, China), was adept at delivering light across a frequency spectrum of 20 Hz to 40 Hz. Control over these frequencies was achieved through a bespoke function signal generator. We calibrated the light intensity at 200 lux, a level determined to stimulate visual pathways effectively without inducing discomfort or potential harm to the subjects. Utilizing MATLAB software, we crafted flicker patterns that allowed the randomization of frequencies within the defined range, thereby facilitating a nuanced exploration of their respective impacts on neuronal activation and the alleviation of fatigue symptoms.

There were two modes of flicker stimulation, specifically at 40 Hz and 20–30 Hz. The 20–30 Hz mode involved light flickering that varied every second, while the 40 Hz mode provided constant frequency stimulation. During the flicker stimulation, normal room lighting was maintained. The flicker stimulation was conducted at 9:00 am and lasted for 1 h, during which time the rats were continuously exposed to the intervention.

### 2.7 Y-maze experiment

After the flicker stimulation, we conducted a Y-maze experiment on each group of rats to evaluate the effect of the flash stimulation on alertness, with *n* = 5 per group. The apparatus featured a three-armed horizontal maze, with each arm measuring 33 cm in length, 10 cm in width, and with walls rising to 11 cm. These arms were spaced at an angle of 120° relative to each other, crafted from dark-colored plastic. This design ensured clear observation of the rats’ behaviors, thanks to the contrast between the rats and the maze. The experimental procedure was structured into three distinct phases. During the initial training phase, one of the arms was blocked using a spacer, thus limiting the rats to the remaining two arms where they were free to roam for 10 min. Following a resting period, the second phase exposed the rats to varied visual flicker stimulations for a duration of 60 min. Finally, the third and concluding phase, known as the testing phase, began with the removal of the spacer. This allowed the rats to be placed randomly in any arm, enabling them to move without constraints for 5 min. Throughout this phase, video tracking was utilized to capture data such as the distance each rat traversed and the duration they spent in each arm over the 5-min period. To ensure the integrity of subsequent trials, the maze was meticulously cleaned using 75% ethanol after each trial, eliminating any residual scents that could influence future sessions.

### 2.8 Immunohistochemistry

Following the Y-maze test, rats in the 40 Hz and random flicker stimulation groups received an additional 60 min of flicker stimulation. Subsequently, three rats from each group were deeply anesthetized. The brains were perfused with PBS, then extracted, and initially fixed in paraformaldehyde for 1 day. Following fixation, the brains underwent dehydration in xylene, followed by a descending ethanol solution series. Utilizing a pathological sectioning machine (LEiCA HistoCore MULTICUT, LEICA MI-CROSYSTEMS LIMITED), the brains were sectioned into 30 μm coronal slices. To enable antigen repair, these tissue sections were placed in an autoclave with EDTA antigen repair buffer (pH 9.0) for 3 min. Following natural cooling, the slides were rinsed thrice in PBS (pH 7.4) with gentle shaking, each lasting 3 min. Thereafter, the sections were treated with endogenous peroxidase blocker and left to incubate for 15 min at room temperature, shielded from light. A subsequent wash in PBS (pH 7.4) was conducted three times for 3 min each with shaking. The primary antibody (ab289723, Abcam, 1:100) was introduced to the sections, followed by an overnight incubation at 4°C. The subsequent day involved incubating the working solution with biotin-labeled goat anti-rabbit IgG and horseradish peroxidase-labeled antibodies for 1 h at 37°C. The color development was initiated with 3,3′-diaminobenzidine (DAB) under microscopic observation, resulting in positive sections adopting a brownish-yellow hue. To stop further color development, the sections were swiftly rinsed with tap water. The slides were then counterstained with hematoxylin, dehydrated through a gradient alcohol series (70, 80, 90%), air-dried post-xylene treatment, and finally sealed with neutral resin. In the concluding step, the images were acquired using a panoramic scanner (Pannoramic Scan, 3DHISTECH).

### 2.9 Molecular biology experiment

After the sleep deprivation and light stimulation procedures, whole blood was collected from the rats, with *n* = 5 per group. This blood sample was then centrifuged at 3000 RPM for 10 min, resulting in the isolation of plasma. By employing a competitive enzyme-linked immunosorbent assay, we quantified the concentrations of 5-HT, cortisol, and melatonin. It is imperative to note that all procedures were rigorously followed according to the manufacturer’s instructions. To ensure accuracy and consistency, each sample was analyzed in triplicate.

### 2.10 Statistics

All data analysis was conducted using GraphPad Prism 9 (GraphPad Software, Inc., United States). Unless mentioned differently in the figure legends, data are presented as means ± SEM. To determine differences between datasets, we employed Two-way ANOVA or One-way ANOVA with a subsequent Bonferroni’s *post-hoc* test, the student’s paired *t*-test, and the paired Wilcoxon test for nonparametric data. Levels of statistical significance were defined as *p < 0.05, **p < 0.01, ***p < 0.001, and ****p < 0.0001.

## 3 Results

### 3.1 A 20–30 Hz random visual flicker enhances resonant frequency neural activity

**Figure 2:**
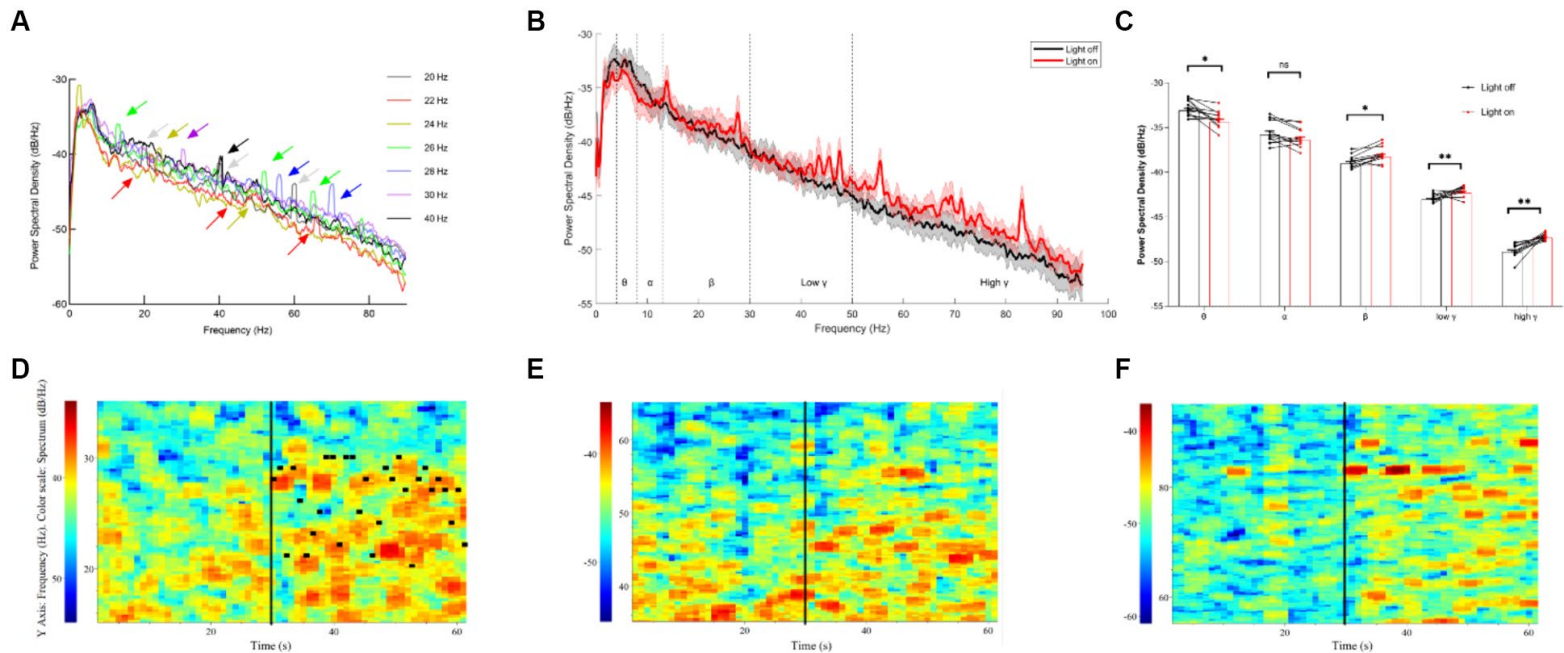
LFP results of the thalamus under different visual flickers. **(A)** PSD of thalamus LFP under flicker stimuli of signal frequency; the arrows indicate the peaks formed by entrainment. **(B)** PSD of thalamic LFPs before and after a 20–30 Hz visual flicker. Colored lines indicate the population average of different conditions, and shaded areas indicate standard error, *n* = 12. **(C)** The mean power histogram of θ, α, β, low γ, and high γ frequency bands before and after a 20–30 Hz visual flicker, T test, *n* = 12. **(D–F)** An example LFP frequency spectrum heatmap under 20–30 Hz visual flicker for β, low γ, and high γ frequency bands. The black vertical lines in the figure represent the start time (30 s) of visual flicker stimulation, and the horizontal black lines in D represent the frequency of random stimuli per second.

To investigate the effects of different light flickers on neural oscillations in the thalamocortical pathway, the recording of single units and LFPs (local field potentials) of the thalamus was undertaken. Initially, we noted the entrainment of LFPs in the thalamus under single light flicker stimuli at various frequencies, including 20 Hz, 22 Hz, 24 Hz, 26 Hz, 28 Hz, 30 Hz, and 40 Hz (Figure 2A). It was observed that the power spectral density (PSD) of multiple resonance frequencies—such as the 10 Hz-15 Hz band, the 20–30 Hz band, the 40 Hz-60 Hz band, and the 60 Hz-90 Hz band—at 20–30 Hz showed a gradual increase immediately after the random light flicker stimulation (Figure 2B). Calculations revealed the mean powers in the following frequency bands, which are crucial for brain function (Figure 2C): 4 Hz – 8 Hz (θ), 8 Hz – 12 Hz (α), 12 Hz – 30 Hz (β), 30 Hz – 60 Hz (low γ), and 60 Hz – 90 Hz (high γ). Moreover, significant decreases in the PSD of θ oscillations and significant increases in the PSD of β and γ oscillations were observed during random visual flicker stimulation. Additionally, the LFP frequency spectrum heatmap depicted changes after random visual flicker stimulation, showing a random increase in LFP intensity in the β, low γ, and high γ bands within the 20–30 Hz, 40–60 Hz, and 60–90 Hz ranges, respectively (Figures 2D–F). However, this increased LFP frequency did not align one-to-one with the stimulus frequency (Figure 2D), where the highest PSD did not necessarily correspond to the stimulus frequency per second. Hence, unlike single-frequency flicker stimulation, random flicker with a frequency of 1-s periodic change may not influence neural oscillations through entrainment, and their underlying mechanisms warrant further input and output data collection and analysis. These findings imply that random visual stimuli potentially enhance brain excitability.

**Figure 3:**
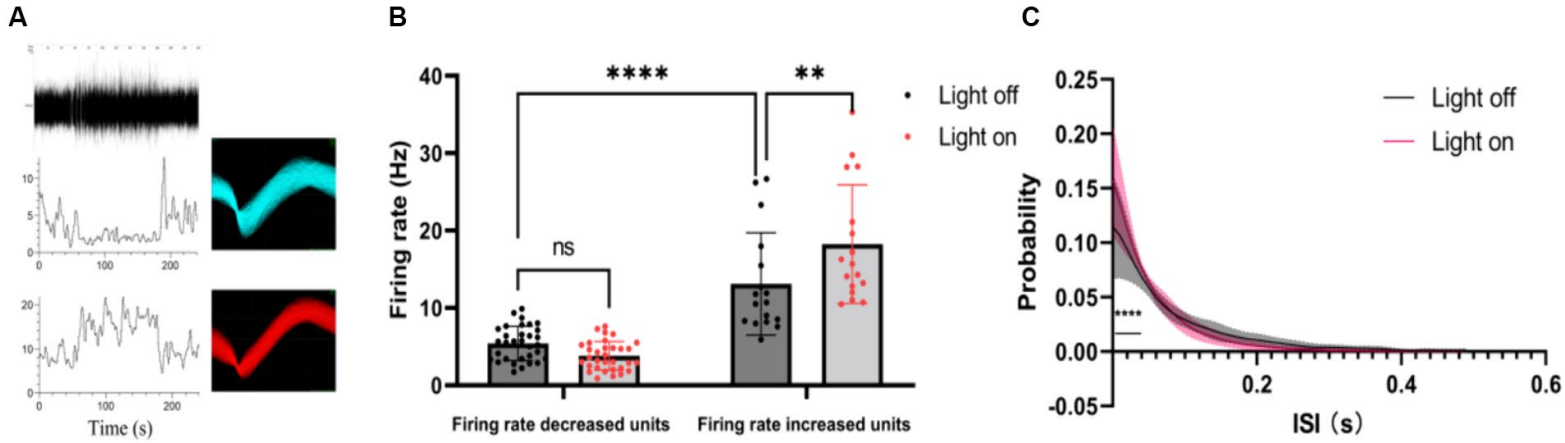
Single unit data of the thalamus under a 20–30 Hz random visual flicker. **(A)** PSTHs of two example units. The upper panel is a raw spike trace of 180 s duration under random visual flicker from 30 s to 150 s. The lower panel shows PSTHs of two example units from the upper raw spike trace. The illustrations on the right are spike waveforms identified using the two example units. **(B)** The mean firing rate of different kinds of units, two-way ANOVA followed by a Bonferroni’s *post-hoc* test, *n* = 32 for units with decreased firing rates and *n* = 17 for units with increased firing rates. **(C)** Probability of interspike interval (ISI) for firing rate increased units (*n* = 17 from 4 rats, two-sided Wilcoxon rank-sum test); colored lines indicate the population average of different units, and shaded areas indicate standard error.

Furthermore, we classified single-unit data into two groups based on their post-stimulus time histograms (PSTHs): units with an increased firing rate and units with a decreased firing rate (Figure 3A). Our analysis sought to verify whether the mean firing rate differed significantly between these two groups. It was found that the units with increased firing rates exhibited a higher average firing rate than the units with decreased firing rates (Figure 3B). Interestingly, under visual flicker stimulation, the mean firing rate showed a significant increase for the units with an increased firing rate, but this was not observed for the units with a decreased firing rate. Additionally, we analyzed the interspike interval (ISI) probability for the units with an increased firing rate and noted a significant increase during the 0–0.04 s interval under visual flicker stimuli (Figure 3C). These findings indicate that visual flicker stimuli not only enhance brain excitability but also improve spike timing precision, potentially facilitating visual information processing.

### 3.2 A 20–30 Hz random visual flicker enhances alertness in rats

To investigate whether light stimulation enhances vigilance in sleep-deprived rats, we used the Y-maze test to evaluate the effects of 40 Hz and 20–30 Hz random visual flicker on their alertness. The results indicated significant differences between the four groups in average speed (*F*_(3, 16)_ = 4.21, *p* = 0.02) and total distance (F _(3, 16)_ = 4.67, *p* = 0.02). Specifically, compared to the Sleep Deprivation group, the 40 Hz group showed significant increases in both average speed and total distance (*p* < 0.05). Additionally, significant differences were observed in the number of novel arm entries (F _(3, 16)_ = 10.74, *p* < 0.001), distance traveled in the novel arm (F _(3, 16)_ = 19.83, *p* < 0.0001), and time spent in the novel arm (F _(3, 16)_ = 11.42, *p* < 0.001). Compared to the Control group, the Sleep Deprivation group showed significant reductions in the number of entries into the novel arm, distance moved, and time spent moving (*p* < 0.001). However, light stimulation post-sleep deprivation (either 40 Hz or 20–30 Hz) significantly enhanced the number of entries into the novel arm, distance traveled, and time spent moving (*p* < 0.05). These findings suggest that flicker stimulation not only enhances motor abilities but also improves learning and memory in rats, likely due to increased alertness induced by the flicker stimulation.

### 3.3 A 20–30 Hz random visual flicker activated multiple alertness-related brain regions

**Figure 4:**
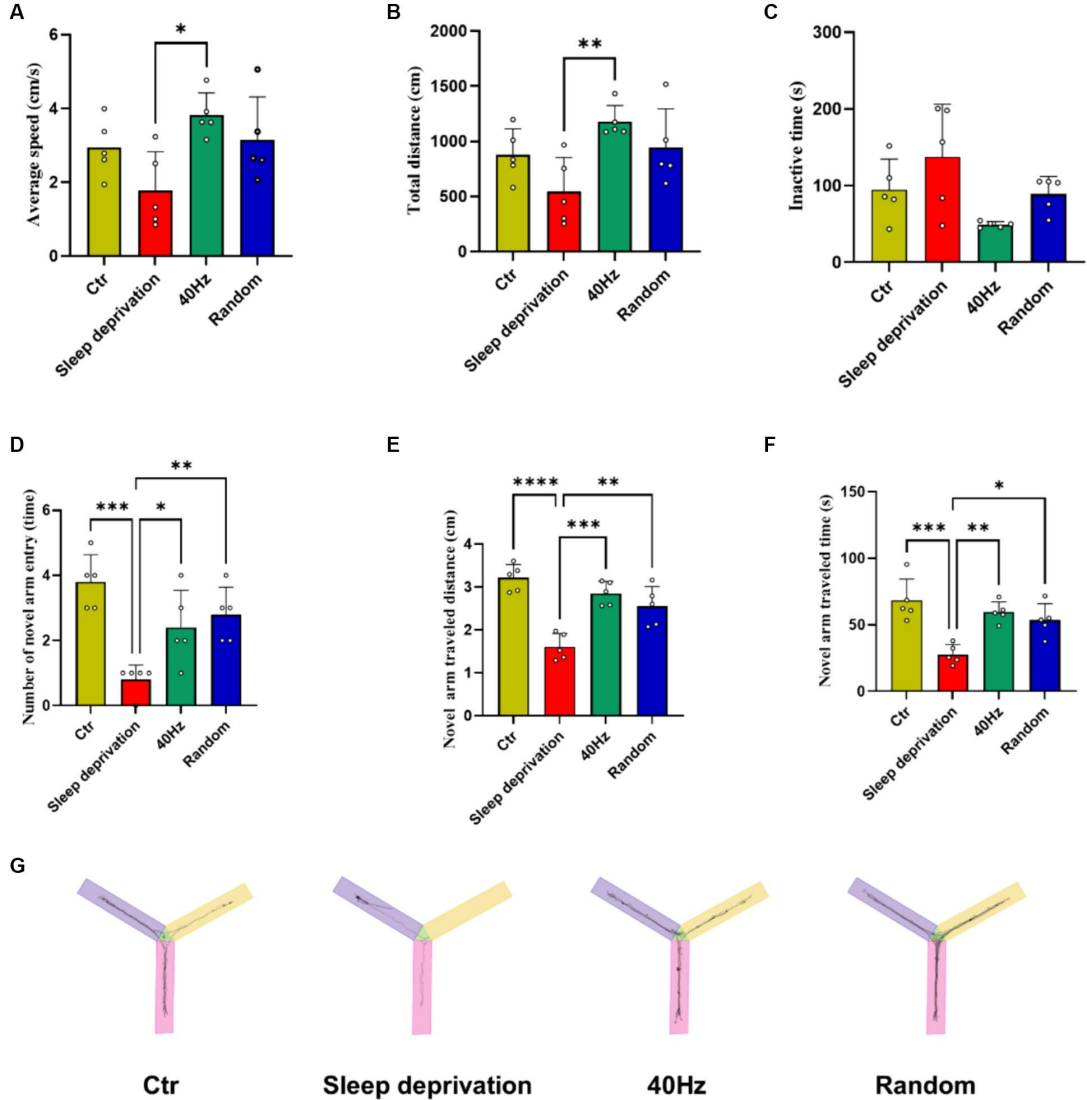
The Effects of 20–30 Hz random visual flicker and 40 Hz visual flicker on alertness in rats. **(A,B)** Demonstration of how light stimulation enhances both the mean speed and distance traveled by sleep-deprived rats in the Y-maze test. **(C)** Light stimulation’s effect in reducing the time spent by sleep-deprived rats in the Y-maze test. **(D)** Illustration of the increase in entries into the new alternative arm in the Y-maze test for sleep-deprived rats due to light stimulation. **(E)** Depiction of how light stimulation augments the distance traveled into the novel alternative arm in the Y-maze test in sleep-deprived rats. **(F)** Examination of the effect of light stimulation on increasing the time spent in the novel arm in the Y-maze test for sleep-deprived rats. **(G)** Trajectory plots of rats in the Y maze after light stimulation exposure for sleep-deprived rats, providing a comprehensive visualization of the effects. **(A,C,E,F)** One-way ANOVA followed by a Bonferroni’s *post-hoc* test, (B, D) non-parametric tests, *n* = 5. *p < 0.05, **p < 0.01, ***p < 0.001, and ****p < 0.0001.

To examine the impact of light flicker on alertness in sleep-deprived rats, we exposed them to normal light alongside specific flicker stimulation, mindful of the masking behavior phenomenon where light exposure typically induces immediate arousal in diurnal animals while promoting sleep in nocturnal animals (Shuboni and Yan, 2010). For the random group, random flicker stimulation at 20–30 Hz was used, while the 40 Hz group received constant 40 Hz flicker stimulation. Given that c-FOS is known to respond uniquely to light stimulation (Beaule et al., 2004) To examine the impact of light flicker on alertness in sleep-deprived rats, we exposed them to normal light alongside specific flicker stimulation, mindful of the masking behavior phenomenon where light exposure typically induces immediate arousal in diurnal animals while promoting sleep in nocturnal animals. For the random group, random flicker stimulation at 20–30 Hz was used, while the 40 Hz group received constant 40 Hz flicker stimulation. Given that c-FOS is known to respond uniquely to light stimulation (Figure 4).

**Figure 5:**
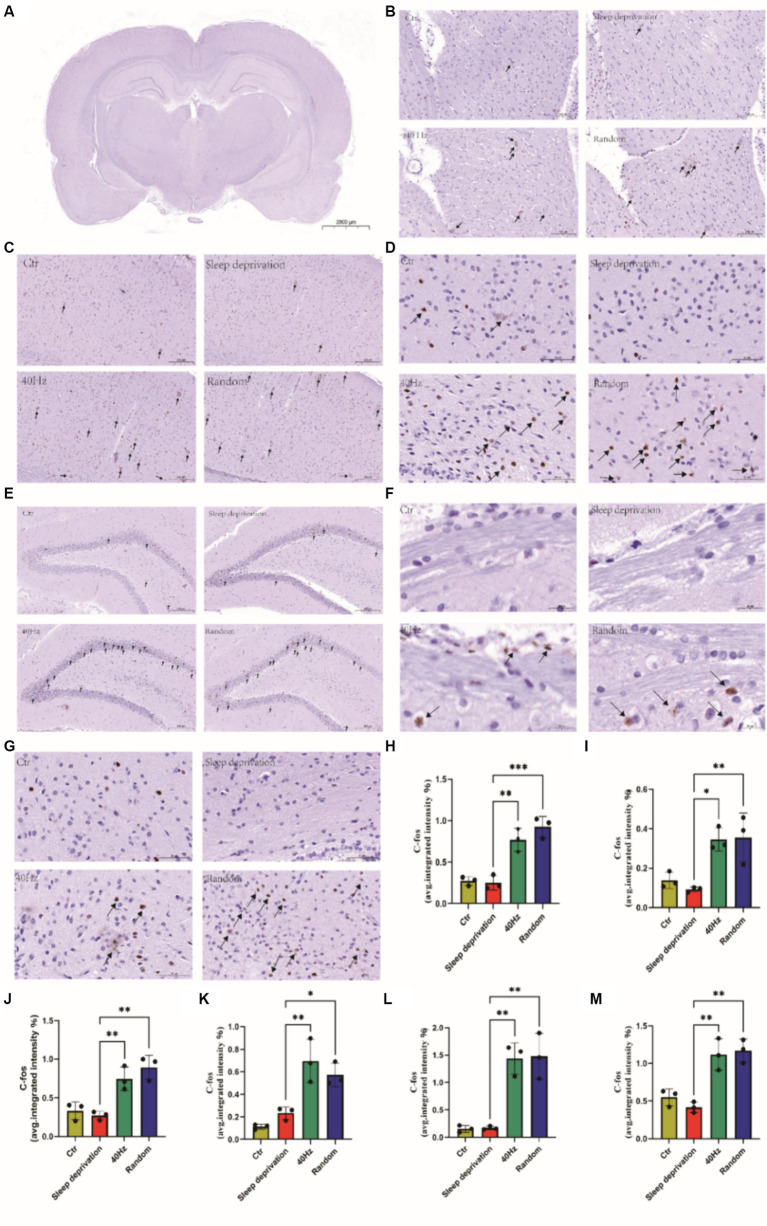
Assessment of the impact of different visual flickers on brain c-FOS expression. Representative immunohistochemical staining (IHC) of c-FOS in the brain is shown. Key regions explored include the following: **(A)** Expression of c-FOS across the whole brain, Scale bar = 2000 μm. **(B)** c-FOS expression in the DLG and VLG brain regions. Scale bar = 100 μm. **(C)** Variations in c-FOS in the V1 brain region, Scale bar = 200 μm. **(D)** c-FOS alterations in the VLPO region of the brain, Scale bar = 50 μm. **(E)** c-FOS changes in the brain’s HPC region, Scale bar = 200 μm. **(F)** c-FOS shifts in the SC region of the brain, Scale bar = 20 μm. **(G)** C-FOS transformations in the SCH region of the brain, Scale bar = 50 μm. **(H)** Regions of the DLG and VLG brain marked with c-FOS IHC staining. Subsequent quantitative analyses were conducted on the IHC-stained regions as follows: **(I)** c-FOS in the V1 brain region. **(J)** c-FOS in the VLPO brain region. **(K)** c-FOS in the HPC brain region. **(L)** c-FOS in the SC brain region. **(M)** c-FOS in the SCH brain region. *p < 0.05, **p < 0.01 and ***p < 0.001.

The results indicated significant changes in c-FOS expression in several brain regions, including the dorsal lateral geniculate nucleus (DLG), ventral lateral geniculate nucleus (VLG), primary visual cortex (V1), ventrolateral preoptic nucleus (VLPO), hippocampus (HPC), superior colliculus (SC), and suprachiasmatic nucleus (SCH; *F*_(3, 8)_ = 11.07, *p* < 0.01 for DLG and VLG; F _(3, 8)_ = 31.54, *p* < 0.001 for V1; F _(3, 8)_ = 17.65, *p* < 0.001 for VLPO; F _(3, 8)_ = 17.60, *p* < 0.001 for HPC; F _(3, 8)_ = 25.91, *p* < 0.001 for SC; F _(3, 8)_ = 20.74, *p* < 0.001 for SCH). Furthermore, compared to the sleep deprivation group, the 40 Hz and random groups exhibited significantly higher levels of c-FOS in the DLG, VLG, VLPO, HPC, SC, and SCH (*p* < 0.01; Figure 5). These findings suggest that flicker stimulation, whether constant at 40 Hz or random within 20–30 Hz, may enhance alertness by influencing brain regions involved in visual processing and sleep–wake regulation, potentially providing a novel approach to addressing alertness through sensory stimulation. This reflects the dual role of light in modulating behavior differently across species, a consideration that enriches our understanding of light’s neurophysiological impact.

### 3.4 A 20–30 Hz random visual flicker has no effect on fatigue recovery in rats

**Figure 6:**
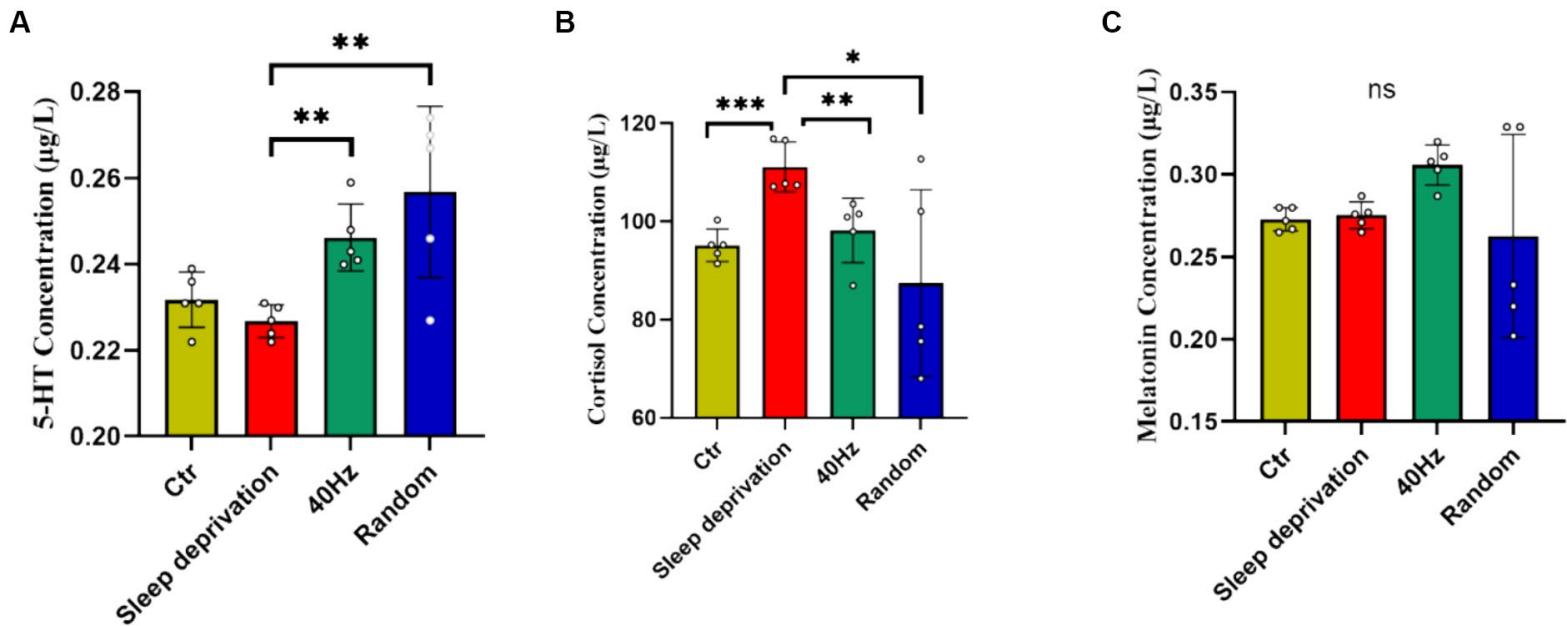
The impact of 20–30 Hz random visual flicker and 40 Hz visual flicker on modulating plasma 5-HT and cortisol levels. **(A)** Quantification of plasma 5-HT levels in light-stimulated sleep-deprived rats as determined by ELISA. **(B)** Quantification of plasma cortisol levels in light-stimulated sleep-deprived rats as determined by ELISA. **(C)** Quantification of plasma melatonin levels in light-stimulated sleep-deprived rats as determined by ELISA. Non-parametric tests, *n* = 5. *p < 0.05, **p < 0.01 and ***p < 0.001.

5-HT, cortisol, and melatonin are crucial neurochemicals intricately involved in central fatigue, sleep regulation, and the body’s response to stress. An aberration in their levels during sleep deprivation can detrimentally impinge on mood and mental disposition (Kang et al., 2021). To investigate the influence of light flicker stimulation on fatigue recovery in sleep-deprived rats, we analyzed the levels of 5-HT, cortisol, and melatonin in rat plasma following a 60-min light stimulation session. The results indicated significant differences across the four groups in plasma levels of 5-HT (*F*_(3, 16)_ = 7.314, *p* < 0.01) and cortisol (F _(3, 16)_ = 4.409, *p* < 0.05). Compared to the sleep deprivation group, both modes of flicker stimulation significantly increased plasma 5-HT levels (*p* < 0.01) and significantly decreased cortisol levels (*p* < 0.05; Figure 6). However, there were no significant differences in plasma melatonin levels following the flicker stimulation. These results suggest that while flicker stimulation may alter neurochemical levels associated with stress and mood, it does not significantly alleviate the sensation of fatigue in the rats.

## 4 Discussion

Neuroimaging studies abound with evidence that the thalamus consistently springs into action when exposed to light during cognitive tasks (Perrin et al., 2004; Vandewalle et al., 2006, 2007). Zooming into specifics, the dorsal and posterior nuclei, particularly encompassing the pulvinar nucleus, are pivotal in intertwining alertness with cognition in humans (Coull et al., 2004; Vandewalle et al., 2009). Another layer to this is that the surge in thalamic activity, consequent to light exposure, is directly correlated with an uptick in subjective alertness (Vandewalle et al., 2009). This correlation concretely establishes that modulating thalamic neural activity is a linchpin in steering alertness. Delving into our findings, we discerned that a singular light flicker stimulus has the potency to usher in entrainment of neuronal oscillations within the thalamus, a revelation echoing earlier studies (Adaikkan et al., 2019; Adaikkan and Tsai, 2019). Furthermore, a light flicker stimulus with a dynamic frequency, changing every second, was observed to amplify neural oscillations within the respective frequency bandwidth. Synthesizing these outcomes, it becomes clear that random light flicker could potentially ameliorate brain fatigue. However, there is a twist: in contrast to a singular light flicker stimulation, the application of random light flicker, when sequenced on a one-second interval, did not manifest any discernible entrainment of neural oscillation. The foray into computational modeling posits that thalamic neural oscillation behaves in a state-contingent manner, imbibing nonlinear traits (Li et al., 2017, 2019). This complexity could elucidate the challenge in spotting overt entrainment when the light flicker is sustained at a uniform frequency for a mere second. Furthermore, the amassed Local Field Potential (LFP) data could be confounded by random noise (Yang et al., 2021), offering a rationale for the observed trend.

Subsequently, we explored the effects of light flicker (40 Hz light flicker and 20–30 Hz random light flicker) on the recovery of alertness in rats after sleep deprivation, particularly focusing on the roles of the thalamus and hippocampus. The thalamus, as the central hub for brain information relay, and the hippocampus, crucial for memory formation and spatial navigation, play vital roles in maintaining and regulating alertness. Our findings indicate that specific frequencies of light flicker significantly enhance the performance of sleep-deprived rats in the Y-maze test, suggesting that light stimulation is beneficial for enhancing alertness. The study found that light stimulation can directly affect neural activity in the brain through photoreceptive cells in the retina, where intrinsically photosensitive retinal ganglion cells (ipRGCs), sensing light through the photopigment melanopsin, relay information to brain areas closely related to the biological clock and alertness regulation, such as the thalamus and hippocampus (Vandewalle et al., 2009; Schmidt et al., 2011). Our data support these findings, confirming the hypothesis that light flicker activates the thalamus and hippocampus through the visual transmission pathway, thereby promoting recovery from central fatigue. Additionally, the interaction between the thalamus and hippocampus plays a complex role in regulating alertness and cognitive functions. The thalamus not only acts as a hub in sensory information processing but also influences memory and learning through its connections with the hippocampus (Jones, 2003). Light flicker stimulation may enhance thalamic activity, indirectly affecting hippocampal function, and thus promoting recovery from central fatigue in a state of sleep deprivation. The specific mechanisms of action still require further molecular and electrophysiological studies for clarification. Furthermore, we also noted that light flickering has a positive effect in alleviating circadian rhythm disruptions in APP/PS1 mice (Xing et al., 2019; Yao et al., 2020). This function may be related to the light stimulation’s ability to alter activity and functional connectivity in core brain areas associated with memory (Lin et al., 2021). This is primarily manifested by improvements in mitochondrial function, reductions in cell apoptosis, and increases in the expression of synaptic proteins post-light flicker (Park et al., 1985), where microglia may play a significant role. Studies have found that light flicker can regulate the immune functions of microglia and has a good regulatory effect on brain inflammation (Venturino et al., 2021). These results suggest that both 40 Hz light flicker and 20–30 Hz random light flicker have a positive effect on the recovery from central fatigue in rats.

Furthermore, we observed that light flicker effectively activates regions in the SD rat brain such as the DLG, VLG, VLPO, HPC, SC, and SCH, as evidenced by significantly increased levels of c-FOS expression in these areas. Notably, previous research has found that sleep deprivation significantly elevates c-FOS expression in the rat brain cortex (Basheer et al., 1998). Our findings indicate that 72 h of sleep deprivation does not significantly alter c-FOS expression levels in the rat brain’s DLG, VLG, VLPO, HPC, SC, and SCH regions. This phenomenon may relate to differential responses of c-FOS in various brain regions to sleep deprivation. Additionally, we noted that following sleep deprivation, c-FOS expression levels in the rat brain cortex could also drastically decrease (Basheer et al., 1998). In our experiments, the control group rats experienced 1 h of normal light stimulation after sleep deprivation, which might also significantly reduce brain c-FOS levels.

5-HT, cortisol, and melatonin all significantly impact central fatigue (Parry et al., 2018; Oikonomou et al., 2019; Kang et al., 2021; Yap et al., 2024). Our research found that light flicker stimulation significantly increases serum 5-HT levels and decreases cortisol levels in rats. Additionally, there is a tendency for melatonin levels to rise after 40 Hz light flicker stimulation. These results suggest that light flicker stimulation may increase the sensation of fatigue in sleep-deprived rats. We hypothesize that light flicker stimulation might overcome fatigue by enhancing alertness, but further experiments are needed to verify this.

### 4.1 Limitations

This study, while presenting promising results on the effects of light flicker stimulation on alertness and cognitive function recovery in sleep-deprived rats, is not without limitations. First, the findings are based on a specific animal model, and thus, caution must be exercised when extrapolating these results to humans. The biological and neurological responses to light stimulation can vary significantly across species, and human studies are necessary to validate these results. Second, the study utilized only two specific frequencies of light flicker (40 Hz and 20–30 Hz random), limiting our understanding of the potential broader spectrum of effective flicker frequencies that could be relevant for therapeutic purposes. Further research exploring a wider range of frequencies and their physiological impacts is essential to fully harness the therapeutic potential of light flicker stimulation. Third, while we measured changes in hormonal levels and neural activation as indicators of fatigue recovery and enhanced alertness, these proxies may not fully capture the complex physiological and psychological state of sleep deprivation and recovery. More comprehensive assessments that include additional biomarkers and subjective measures of mood and cognitive function are needed to provide a fuller picture. Lastly, the duration of the light flicker exposure and the follow-up period were relatively short. Longer-term studies are needed to assess the sustainability of the cognitive benefits and any potential long-term adverse effects of chronic light flicker exposure. In conclusion, while these findings contribute valuable insights into the potential benefits of light flicker stimulation, they also highlight the necessity for additional studies to overcome these limitations and further clarify the clinical relevance of this intervention.

## 5 Conclusion

This study has provided compelling evidence that visual light flicker stimulation, at frequencies of 40 Hz and 20–30 Hz random flicker, can enhance alertness and aid in the recovery of cognitive functions in sleep-deprived rats. Through a combination of behavioral, neurophysiological, and molecular approaches, we have demonstrated that light flicker can activate crucial brain regions associated with alertness and cognitive processes, such as the thalamus and hippocampus, and modulate neurochemical pathways that are vital for stress response and mood regulation. The significant increase in c-FOS expression and the normalization of cortisol and serotonin levels in treated rats underscore the potential of targeted light therapy in managing conditions related to sleep deprivation and possibly other cognitive impairments. These findings suggest that flicker stimulation, by enhancing neural activity and adjusting critical neurochemical levels, holds promise as a non-pharmacological approach to bolster cognitive resilience and alertness. However, it is important to acknowledge the limitations of this study, including its applicational bounds in human models, the narrow range of frequencies tested, and the short-term nature of the interventions. Future research should aim to explore the effects of a broader range of flicker frequencies over extended periods and in diverse populations, including human subjects, to ascertain the clinical viability and safety of this intervention. In conclusion, while this research marks a significant step toward understanding the cognitive and neurophysiological impacts of light flicker stimulation, it also opens avenues for further studies to explore its practical applications in clinical settings and everyday life, potentially revolutionizing approaches to cognitive enhancement and fatigue management.

## Data availability statement

The raw data supporting the conclusions of this article will be made available by the authors, without undue reservation.

## Ethics statement

The animal study was approved by the Institutional Animal Care and Use Committee (IACUC) of the Chinese Academy of Military Medical Science. The study was conducted in accordance with the local legislation and institutional requirements.

## Author contributions

KW: Conceptualization, Data curation, Funding acquisition, Writing – original draft, Writing – review & editing. KC: Conceptualization, Data curation, Formal analysis, Validation, Writing – original draft, Writing – review & editing. ZW: Conceptualization, Data curation, Writing – original draft, Writing – review & editing. TW: Conceptualization, Data curation, Writing – original draft, Writing – review & editing. AW: Investigation, Methodology, Writing – original draft, Writing – review & editing. XG: Conceptualization, Data curation, Investigation, Writing – original draft, Writing – review & editing. YQ: Software, Supervision, Writing – original draft, Writing – review & editing. YZ: Conceptualization, Data curation, Formal analysis, Writing – original draft, Writing – review & editing. YG: Conceptualization, Data curation, Writing – original draft, Writing – review & editing. BC: Funding acquisition, Investigation, Project administration, Resources, Writing – original draft, Writing – review & editing. MZ: Data curation, Formal analysis, Investigation, Methodology, Project administration, Resources, Writing – original draft, Writing – review & editing.
